# Advanced 3D echocardiographic assessment of the aortic valve and aortic root complex: a repair-oriented perspective

**DOI:** 10.1093/ehjimp/qyag136

**Published:** 2026-08-07

**Authors:** Tommaso Viva, Corrado Fiore, Raluca Dulgheru, Daniele Andreini, Mattia Glauber, Patrizio Lancellotti

**Affiliations:** Department of Clinical Cardiology and Cardiovascular Imaging, IRCCS Ospedale Galeazzi-Sant'Ambrogio, Via Cristina Belgioioso 173, Milan 20157, Italy; Department of Cardiology, GVM Care & Research, Città di Lecce Hospital, Lecce, Italy; Department of Cardiology, GIGA Institute Cardiovascular Science & Metabolism, CHU Sart Tilman, Sart Tilman, University of Liège Hospital, Domaine Universitaire du Sart Tilman, Liège B.35–4000, Belgium; Department of Clinical Cardiology and Cardiovascular Imaging, IRCCS Ospedale Galeazzi-Sant'Ambrogio, Via Cristina Belgioioso 173, Milan 20157, Italy; Department of Biomedical and Clinical Sciences, University of Milan, Milan, Italy; Minimally Invasive Cardiac Surgery Unit, IRCCS Ospedale Galeazzi—Sant'Ambrogio, Milan, Italy; Department of Cardiology, GIGA Institute Cardiovascular Science & Metabolism, CHU Sart Tilman, Sart Tilman, University of Liège Hospital, Domaine Universitaire du Sart Tilman, Liège B.35–4000, Belgium

**Keywords:** three-dimensional echocardiography, aortic valve, aortic root complex, bicuspid aortic valve, aortic valve repair

## Abstract

**Aims:**

The aortic valve (AV) and aortic root complex constitute a functional unit, and their integrated anatomical and dynamic assessment is essential for the management of AV disease. This review highlights the role of three-dimensional echocardiography (3DE) in the comprehensive evaluation of AV disease and its contribution to therapeutic decision-making.

**Methods and results:**

Owing to close collaboration with cardiac surgeons, 3DE has significantly expanded the diagnostic information provided by imaging cardiologists and has become a key tool for guiding therapeutic strategies. In patients with significant aortic regurgitation (AR), detailed anatomical assessment of the cusps enables characterization of valve morphology and tissue quality, while multiplanar reconstruction provides accurate measurements of the virtual basal ring, coronary ostial height, and other key anatomical structures. Quantitative assessment of cusp geometry, including effective, geometric, and coaptation heights, allows precise identification of AR mechanisms, thereby facilitating repair-oriented surgical planning. In aortic stenosis (AS), 3DE improves evaluation of discordant or borderline cases through enhanced assessment of the left ventricular outflow tract and direct planimetric measurement of AV area. In addition, 3DE provides complementary information for procedural planning of both transcatheter and surgical AV replacement, particularly in patients with contraindications to computed tomography.

**Conclusion:**

Three-dimensional echocardiography, particularly transoesophageal 3DE, has become an essential imaging modality for integrating anatomical and functional information. It improves diagnostic accuracy, supports individualized therapeutic planning, and plays a pivotal role in promoting a repair-oriented approach to the management of aortic valve disease.

## Graphical Abstract

**Graphical Abstract:**
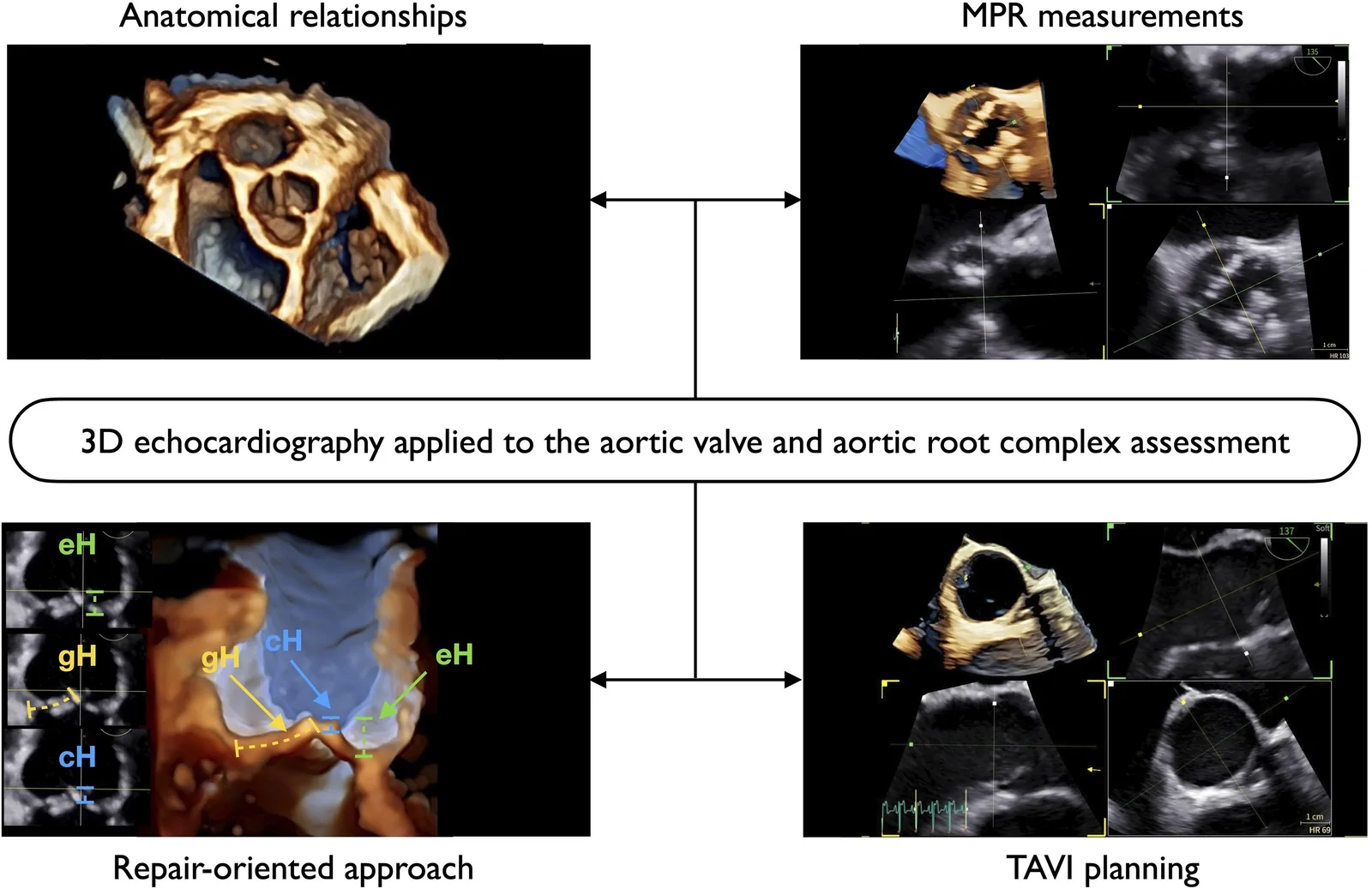

## Introduction

The anatomy and pathophysiology of the aortic valve (AV) and aortic root complex often raise questions and challenges in the clinical practice of cardiologists specialized in imaging. The anatomopathological knowledge of this anatomical region, shared by cardiac surgeons, has progressively enriched the information that imaging specialists can provide through echocardiography. Thus, close collaboration between imaging cardiologists and surgeons has evolved to provide the best possible treatment for patients.^1^ This collaboration has become pivotal with the progressive spread of AV repair and valve-sparing root replacement (VSRR) techniques. The need for a deep and detailed imaging analysis has become essential. At the same time, insights gained from surgery have been translated into imaging applied in the interventional domain, particularly in the pre-procedural assessment for transcatheter aortic valve implantation (TAVI).

The basic principle is that AV and aortic root constitute a functional unit. The integration of anatomical and dynamic information of this entire region allows a comprehensive understanding of disease mechanism, thus supporting appropriate treatment selection. Three-dimensional echocardiography (3DE) represents an extraordinary tool to assess both anatomy and pathophysiology simultaneously. Historically, 3DE has been less widely used for AV than for the mitral valve, because of the lower adoption of valve repair techniques, both surgical and transcatheter, and for the lower quality of 3D images, particularly due to dropout artefacts. Two-dimensional echocardiography has demonstrated a limited diagnostic accuracy in defining the mechanism of aortic regurgitation (AR),^2^ highlighting the essential role of 3DE in the evaluation of the AV and aortic root complex. Transthoracic echocardiography (TTE) is the first-line exam, in which 3DE can be applied to provide additional useful information. Transoesophageal echocardiography (TOE) represents the cornerstone for the detailed assessment of both normal and pathological findings. The general principles of three-dimensional (3D) imaging acquisition and post-processing have been described in previous works.^3–5^ Specific 3D views for the AV and aortic root will be described in the following sections.

The aim of this work is to provide readers with a theoretical and practical approach to 3DE, especially in the transoesophageal setting, applied to the assessment of the AV and aortic root complex, to give the basis for a correct diagnosis and to propose tailored treatment, in particular in terms of valvular repair or valve-sparing surgery.

### 3D echocardiographic anatomy

A complete anatomical description of the AV, aortic root, and tubular ascending aorta (TAA) has to be done to understand dysfunction, as they work as a functional unit. The aortic root complex indeed acts as a supporting structure of the AV, being extended from the left ventricular outflow tract (LVOT) to the sinotubular junction (STJ).^6,7^ The following description will be presented through 3D TOE images, as this represents the best modality to appreciate anatomical structures and relationships.

**Figure 1:**
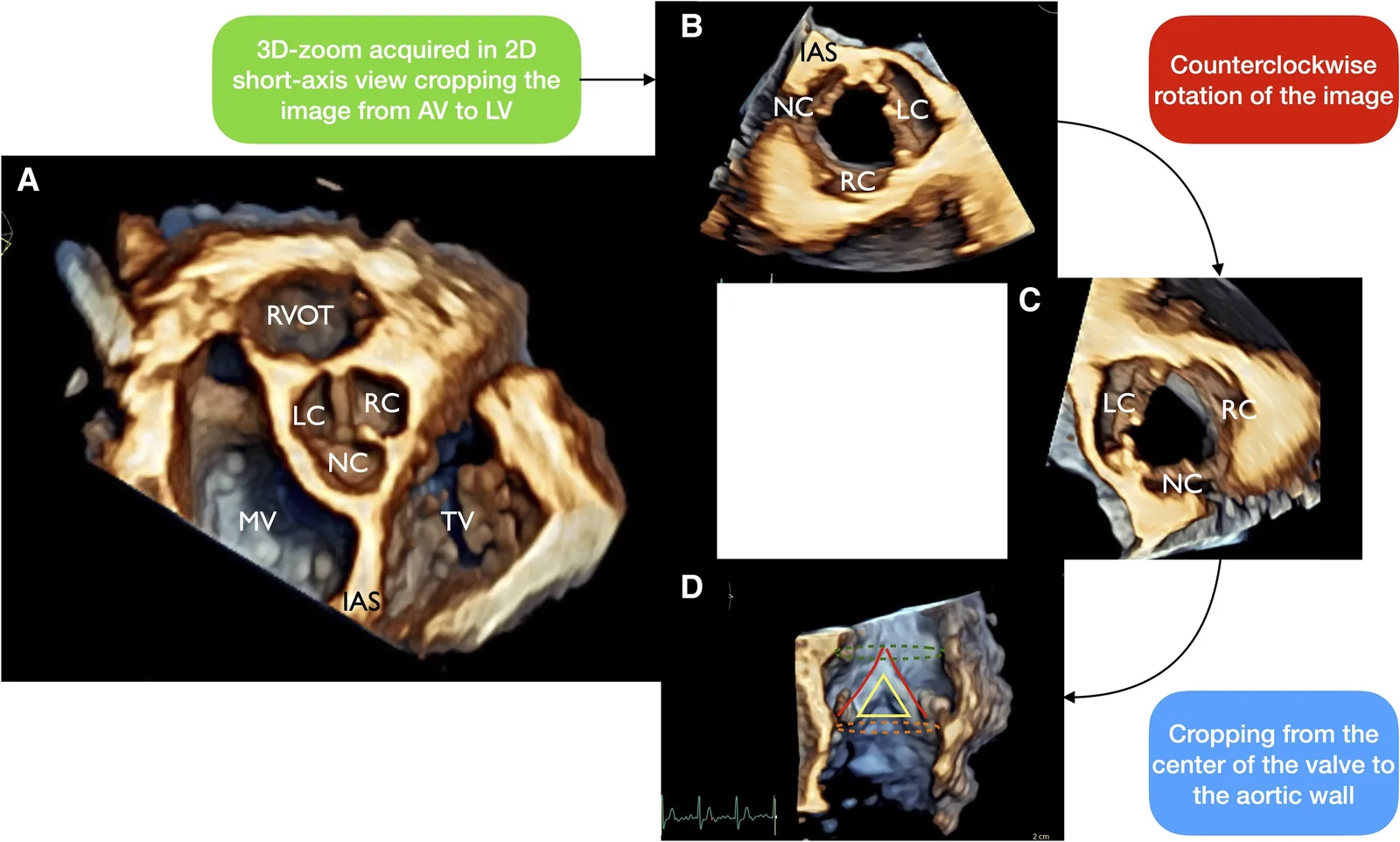
3D TOE acquisition and orientation of the aortic valve (AV). Panel *A*: 3D full-volume acquisition of the base of the heart cropping the image from the base to the apex. This figure illustrates the anatomical relationship of the AV with the surrounding structures: right ventricular outflow tract, interatrial septum, mitral valve and tricuspid valve. Panel *B*: 3D ‘en face’ of the AV acquired using ‘3D zoom-mode’ and oriented as in the mid-oesophageal short-axis view. Inclusion of portions of the surrounding structures within the crop box facilitates a correct 3D orientation. Panel *C*: 3D ‘surgical view’ orientation of the AV obtained by counter-clockwise rotation of the previous image. Panel *D*: 3D ‘long-axis’ view rotated counter-clockwise with zooming on the valve. This view magnifies the interleaflet triangles (yellow triangle), and the ventriculo-aortic physiological junction, corresponding to the attachment of each cusp to the aortic wall (called ‘surgical annulus’, red lines), located between the plane of the virtual basal ring (orange dotted line) and the sinotubular junction (green dotted line).

Understanding the position of the aortic root within the chest and its relation to adjacent structures is important. This can be easily shown from a 3D full volume acquisition of the base of the heart (*Figure 1A*). The aortic root complex is located posteriorly and to the right of the right ventricular outflow tract (RVOT), which is related to the left and right coronary sinuses. The non-coronary sinus is the most distant from the RVOT and continues posteriorly and superiorly with the interatrial septum and posteriorly and inferiorly with the anterior and septal leaflets of the tricuspid valve. The left and non-coronary sinuses are separated from the mitral valve by a rectangular sheet of fibrous tissue, known as mitral-aortic curtain.

A ‘3D zoom-mode’ image of the aortic root complex can be acquired from the mid-oesophageal view in either short (approximately 45°) or long (approximately 135°) axis. Imaging ‘from above’ the AV may enhance image quality by avoiding drop-out artefacts due to parallel alignment between the US beam and the aortic cusp’s tissue. Including portions of surrounding structures, such as interatrial septum and RVOT, within the crop box facilitates a correct 3D orientation. This focused acquisition enhances visualization of anatomical details. Cropping the dataset from the aorta to the left ventricle generates the ‘en face aortic’ view, the most familiar 3D perspective. The AV can be oriented as in mid-oesophageal short-axis view (*Figure 1B*). Alternatively, counter-clockwise rotation of the image produces the so-called ‘surgical view’ (*Figure 1C*). This term is derived from the orientation of the aortic root complex in relation to the cardiac surgeon’s perspective when the TAA is opened. In this view, the left coronary cusp is located in the upper left of the screen, the right coronary cusp in the upper right, and the non-coronary cusp in the lower portion. From the aortic view, the sinuses of Valsalva (SoV), the aortic surface of the cusps, and the lines of apposition and coaptation between the cusps can be appreciated. Moreover, this perspective allows accurate identification of the number of commissures (and the presence of raphes, if any) to define the type of valve anatomy. The number of functional commissures is the most important anatomical criterion indicating if a valve is unicuspid, bicuspid, tricuspid, or quadricuspid. When included in the crop box, the origin of the coronary arteries can be visualized within their respective coronary sinuses (in normal anatomy), at or below the level of the STJ.^7^

**Figure 2:**
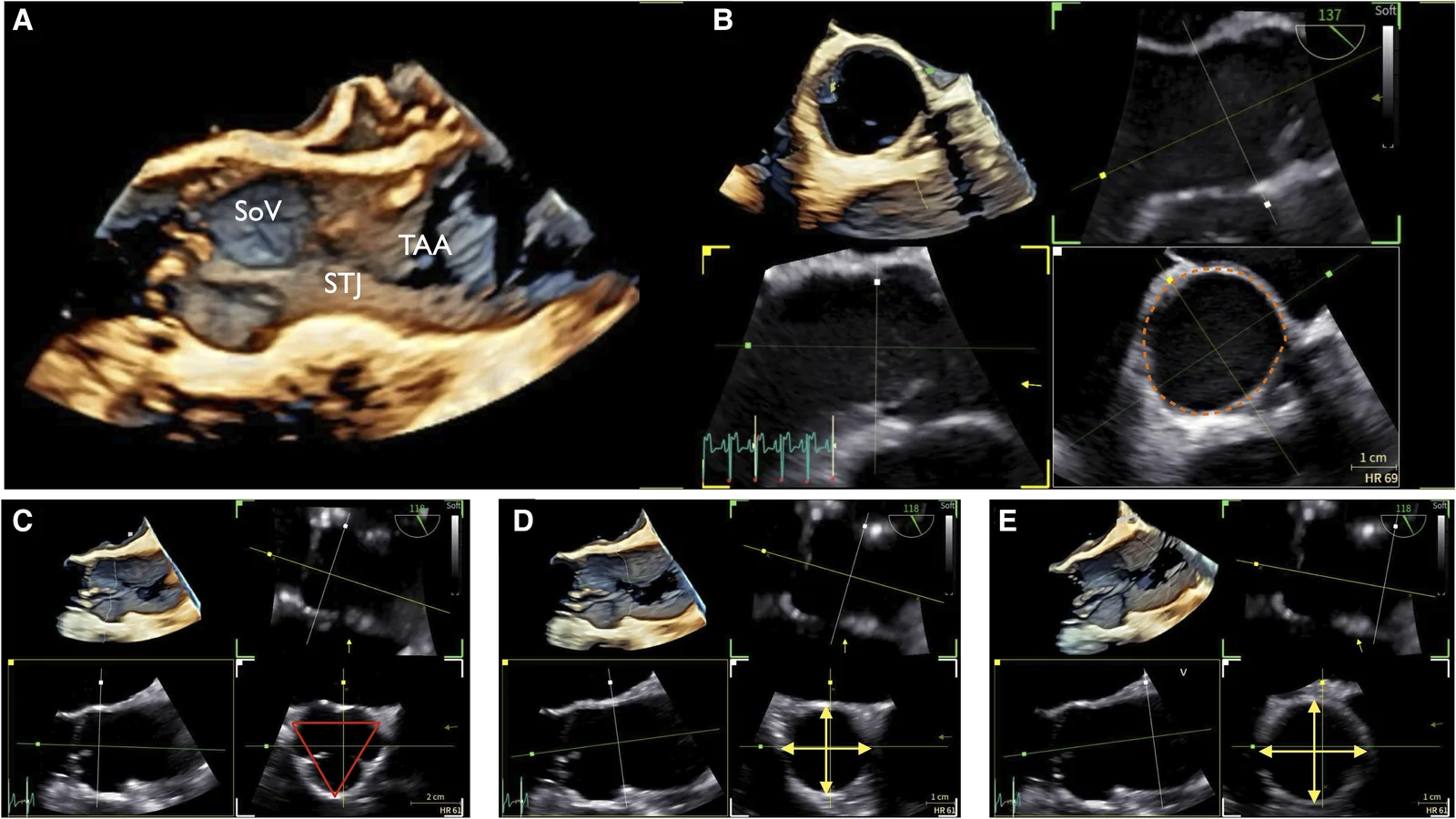
3D TOE dataset acquisition including all the aortic portions. Panel *A*: 3D ‘long-axis’ extending from the left ventricular outflow tract to the tubular ascending aorta (TAA). Panel *B*: Multi-planar reconstruction (MPR) of a 3D dataset showing the virtual basal ring (orange dotted line). Panel *C*: 3D MPR at the sinuses of Valsalva (SoV) level; measurements are shown at the maximal sinus-to-sinus diameter at end-diastole (red lines). Panel *D*: 3D MPR at the sinotubular junction (STJ) level; measurements are shown with yellow lines. Panel *E*: 3D MPR at the TAA level; measurements are shown with yellow lines.

The 3D ‘long-axis’ is obtained either by acquiring the imaging in 2D long-axis view or by cropping the 3D dataset from the centre of the aorta towards the aortic wall (*Figure 1D*). It is typically extended from the LVOT to the TAA. This view can also be displayed in 2D long-axis orientation (*Figure 2A*). In this view, both the aortic and ventricular surfaces of the cusps are visible. Cusp thickness and the presence of tissue abnormalities, such as calcification or fibrosis, can be appreciated. Each cusp has a crescentic coaptation surface on the ventricular side, which allows a perfect coaptation between adjacent cusps. This zone of apposition between cusps is termed *lunula*. At the centre of each cusp there is a small nodular thickening, the so-called *nodule of Arantius,* which enhances coaptation at the centre of the valve, preventing central jets. Each cusp is attached to the aortic root wall in a semilunar fashion. The hinge lines of adjacent cusps meet at the level of the STJ, forming the commissures.^7^ The 3D long-axis view allows to appreciate the height of the commissures, which may vary in case of bicuspid aortic valve (BAV),^8^ and, if present, the length of a raphe. Beneath each commissure, the thin *interleaflet fibrous triangles* are well visualized (*Figure 1D*). The triangle between the left and non-coronary cusps is part of the mitral-aortic curtain, whereas the triangle between the right and non-coronary cusps is incorporated into the interventricular membranous septum.^6,7^ At the level of the proximal portion of each cusp, the ventriculo-aortic junction (VAJ) requires a careful explanation. Firstly, a distinction between physiological and anatomical VAJ is necessary.^9^ The physiological VAJ, corresponding to the attachment of each cusp to the aortic wall, has a ‘crown-like’ shape and is referred to as the ‘surgical annulus’, representing how the cusps are supported by the aortic root complex (*Figure 1D*).^9^ The anatomical VAJ, the junction between ventricular and aortic tissue, does not completely coincide with the physiological VAJ. Posteriorly, the ventricular side is composed of fibrous tissue (part of the mitral-aortic curtain), making the exact transition indistinct; anteriorly, ventricular myocardium extends across the hinge lines of the cusp and is included in the aortic sinuses.^7,9^ This anatomical arrangement highlights that a true annulus, in the strict sense of a complete ring, does not exist.^6,10^ However, in clinical practice and in the imagers’ lexicon, we use the term ‘annulus’ to define a virtual circumference. This echocardiographic annulus, the so-called ‘virtual basal ring’ (VBR), is defined by connecting the nadirs of the cusp hinge lines. Multi-planar reconstruction (MPR) of a 3D dataset can accurately depict the VBR, typically an oval-shaped structure, enabling measurements of diameters, circumference, and area with implications for surgical and interventional procedures (*Figure 2B*).

The ‘ventricular view’ is obtained by cropping the 3D dataset from the left ventricle towards the aorta. This view displays the LVOT, the mitral-aortic curtain, and the ventricular surfaces of the aortic cusps. It is useful for identifying areas of loss of coaptation between the cusps, a cusp restrictive motion or a perforation, as well as the presence of masses/filamentous structures (e.g. vegetations, Lamble excrescences). Visualization of pathological regions can be enhanced using transillumination modality positioning the light source in the aorta.

Finally, a 3D dataset including all the aortic portions (from the SoV to the TAA, *Figure 2A*) enables precise measurements of aortic dimensions using MPR. Measurements at the aortic root level should be performed at the maximal sinus-to-sinus diameter at end-diastole (*Figure 2C*).^11^ Although validation studies are needed, the leading edge-to-leading edge method is recommended in echocardiography,^11^ although some authors proposed inner edge-to-inner edge due to the comparable spatial resolution of 3D TOE with cardiac magnetic resonance and computed tomography (CT).^12^ The use of 3D MPR is particularly valuable in cases of asymmetric dilation of a single sinus, commonly the non-coronary sinus. Measurement of the STJ and the TAA can also be easily obtained using MPR (*Figure 2D, 2E*). In dilated aortic roots, the precise localization of the STJ may be difficult with 2D imaging because of the distorted anatomy, but 3D imaging is extremely precise in identifying the STJ by progressively lowering the MPR plane from the TAA to the commissures of the cusps. The plane intersecting all the commissures is therefore the actual plane of the STJ. In contrast to the VBR, STJ is a true anatomical ring, composed of circumferential fibro-elastic lamellae with localized thickening near the commissures.^6,7,13^ The normal mean STJ diameter is larger than VBR, with an STJ/VBR ratio of approximately 1.2.^14^

### Aortic regurgitation assessed by 3D echocardiography: when geometry defines the mechanism

**Figure 3:**
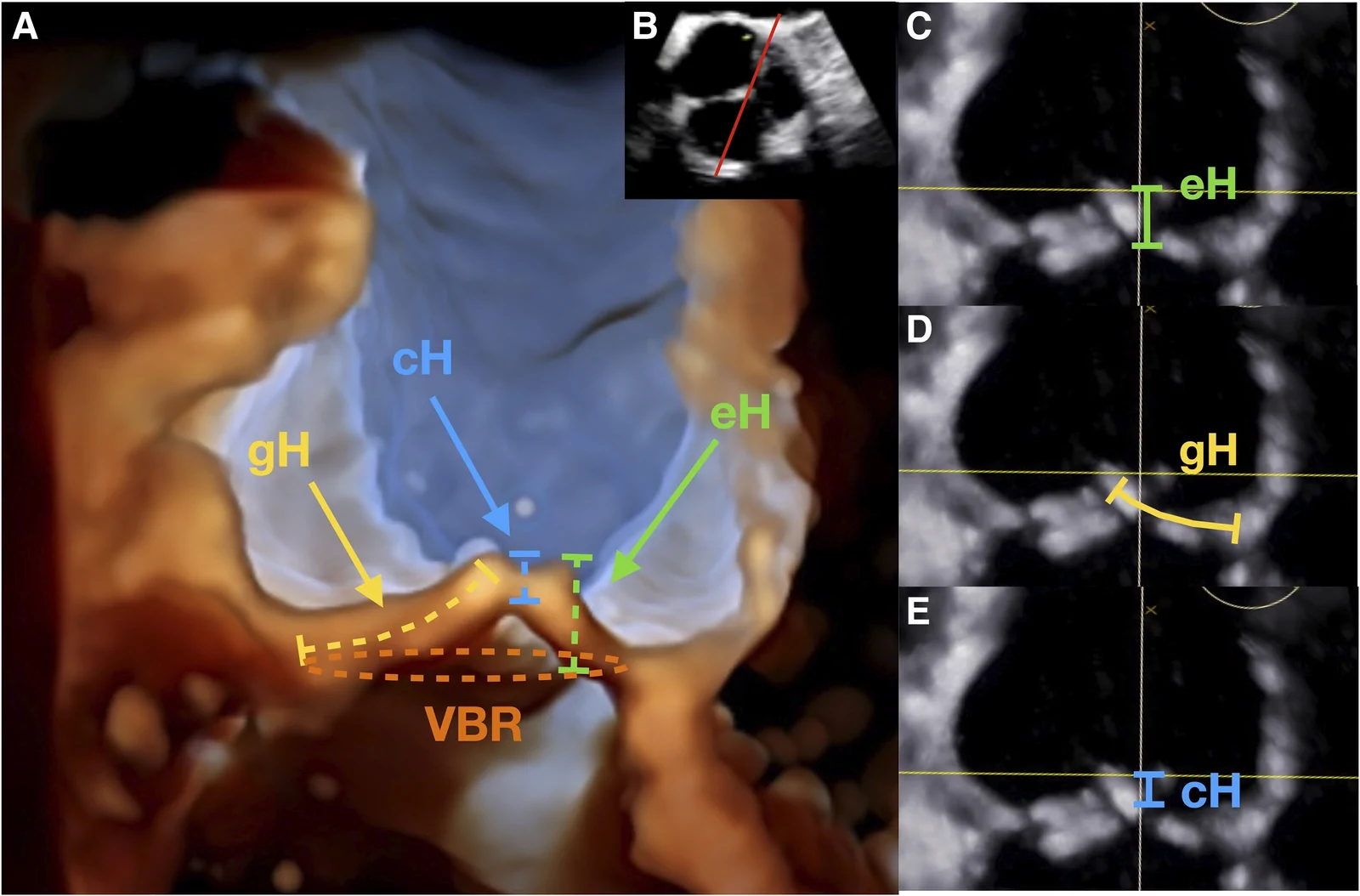
Geometric relationships within the aortic root complex. Panel *A*: 3D ‘long-axis’ view with transillumination modality to highlight a 3D perspective of cusp heights: effective height (green dotted line), coaptation height (light blue dotted line), and geometric height (yellow dotted line). Panel *B*: Short-axis view showing the plane (red line) cutting the cusp in its centre in multi-planar reconstruction (MPR) to correctly measure cusp heights (see following panels). Panel *C*: MPR used to measure effective height (green line). Panel *D*: MPR used to measure geometric height (yellow line). Panel *E*: MPR used to measure coaptation height (light blue line).

Once clarified that the aortic root complex acts as a functional unit, it is easy to understand that the mechanisms of AR may originate from dysfunction/distortion of any of its components.^15^ Analogous to Carpentier’s classification for mitral regurgitation, El Khoury proposed a functional classification for AR based on cusp motion to promote a systematic approach to the AV repair: normal cusp motion with aortic dilatation (Type I), excessive cusp motion/prolapse (Type II), and ‘restrictive’ cusp motion (Type III).^16^ Before describing these mechanisms in detail, it is important to note that a semantic issue within this classification may generate confusion: in Type I AR, cusp motion appears normal, but actually aortic dilatation often causes a functional restrictive systolic and diastolic motion of the cusps (cusp tethering). In contrast, Type III AR is generated by an alteration of the cusp’s tissue (histological change in cusp tissue, namely cusp retraction/lack of tissue) that modifies the coaptation surface of the cusps, and hence AR may induce an organic restrictive cusp motion.^17^ This classification qualitatively defines cusp motion and indirectly reflects cusp morphology. However, since the first concepts introduced by Swans and Clark in 1974 regarding the geometric relationships within the aortic root complex^18^ and subsequently developed from the 1990’s onward,^19–21^ it has become clear that precise quantification and measurement of cusp heights (*Figure 3A*) and their relationship with aortic root complex dimensions allows accurate identification of the AR mechanism, and, more importantly, define the mechanism of AR and guide the feasibility of an AV repair or VSRR (*Table 1*).^19^ Notably, the 2D mid-oesophageal long-axis view allows orthogonal visualization only of the right cusp, whereas the non-coronary and left coronary cusps are visualized through off-axis planes, with a consequent risk of measurement errors in cusp height. Therefore, although 2D echocardiography and colour flow direction may suggest the AR mechanism, they have limited accuracy to define AR mechanism assessed intraoperatively.^2^ For this reason, a significant AR should be routinely approached using 3D TOE with MPR to accurately measure cusp heights and aortic root complex dimensions. The three heights can be measured intraoperatively using a calliper or non-invasively with advanced imaging techniques, such as 3DE or CT.

**Table 1:** Key geometric parameters for aortic valve repair-oriented assessment

Parameter	Definition	Clinical/Surgical Relevance	Normal Value	Critical Thresholds
Effective height (eH)	Orthogonal distance between the virtual basal ring (VBR) and the midpoint of the cusp free margin in end-diastole	Identifies cusp prolapse	≥ 9–10 mm	0 < eH < 9 mm: incomplete prolapse eH < 0 mm: complete prolapse
Geometric height (gH)—tricuspid aortic valve	Maximal length of one cusp measured from the free margin to the nadir of insertion	Reflects the amount of available cusp tissue and repair feasibility	≈ 19–20 mm	Surgical thresholds for repairability: gH ≤ 16 mm
Geometric Height (gH)—non-fused cusp in bicuspid valve	Maximal cusp length of the non-fused cusp	Reflects the amount of available cusp tissue and repair feasibility	≈ 24 mm	Surgical threshold for repairability: gH ≤ 18 mm
coaptation height (cH)	Length of apposition between adjacent cusps during diastole	Marker of valve competence and repair durability	≈ 5 mm	cH < 5 mm indicates reduced cusp coaptation
Virtual basal ring (VBR) diameter	Diameter measured through 3D multi-planar reconstruction connecting the nadirs of cusp insertion	Essential for repair planning	Variable according to body size	VBR ≥ 25 mm A dilated VBR warrants the adoption of an annuloplasty technique
Sinotubular junction (STJ)/VBR ratio	Ratio between STJ diameter and VBR diameter	Assesses root geometry and identifies type I AR mechanisms	≈ 1.2	STJ/VBR >1.2: in favour of type 1a and 1b mechanisms; STJ/VBR <1.2: in favour of type 1c mechanism.
Commissural orientation (BAV)	Angle between the two functional commissures in fused bicuspid aortic valves	Determines repair strategy Symmetric valve predicts freedom from reoperation after repair	160–180° (symmetrical)	Asymmetrical (160°–140°) Very asymmetrical (140°–120°)
Coronary ostial height	Distance between the VBR and coronary ostia	Critical for valve-sparing root replacement and TAVI planning	Variable	—

The effective height (eH) represents the orthogonal distance between the VBR plane and the midpoint of each free margin of the cusps in end-diastole (*Figure 3C*). In other words, it is the height from the VBR plane to each nodule of Arantius and indicates how ‘high’ or ‘low,’ relative to the VBR, each cusp descends in end-diastole. In a competent AV, this distance should ideally be identical in every cusp and no lower than 8–10 mm.^14,19^ For imagers, the term ‘VBR-to-Arantius nodule’ length may be more intuitive than eH, which was defined by cardiac surgeons.

The geometric height (gH) represents the maximal length of the cusp in its mid portion, and it is measured from the midpoint of the free margin to the connection with the aortic wall (the nadir of the cusp) (*Figure 3D*). In other words, it is a measure of how much tissue is available for repair. gH can be measured both in systole and diastole. When image quality is suboptimal, gH may be difficult to measure in diastole, and systolic images may be more adequate. When AV cusps are retracted or damaged, gH is reduced, signalling that tissue quality is poor or there is insufficient tissue for proper repair. For imagers, ‘curved cusp length’ may also be easier to adopt than gH. The normal intraoperative gH is approximately 19–20 mm in a tricuspid aortic valve (TAV) and 24 mm in non-fused cusp of a BAV.^20,22^ The non-coronary cusp has been reported to be slightly longer than the other two.^20^ gH value correlates with body surface area, age, and gender. A weak correlation was also found between gH and root dimensions.^20^ No studies have yet compared intraoperative and 3D MPR measurements.

The coaptation height (cH) is the length of apposition between two cusps in diastole (*Figure 3E*). The measurement should be taken halfway between the midpoint of the free cusp edge and the commissures. It measures approximately 5 mm in a normal AV. Reduction in cH represents the final result of the mechanism causing AR.

**Figure 4:**
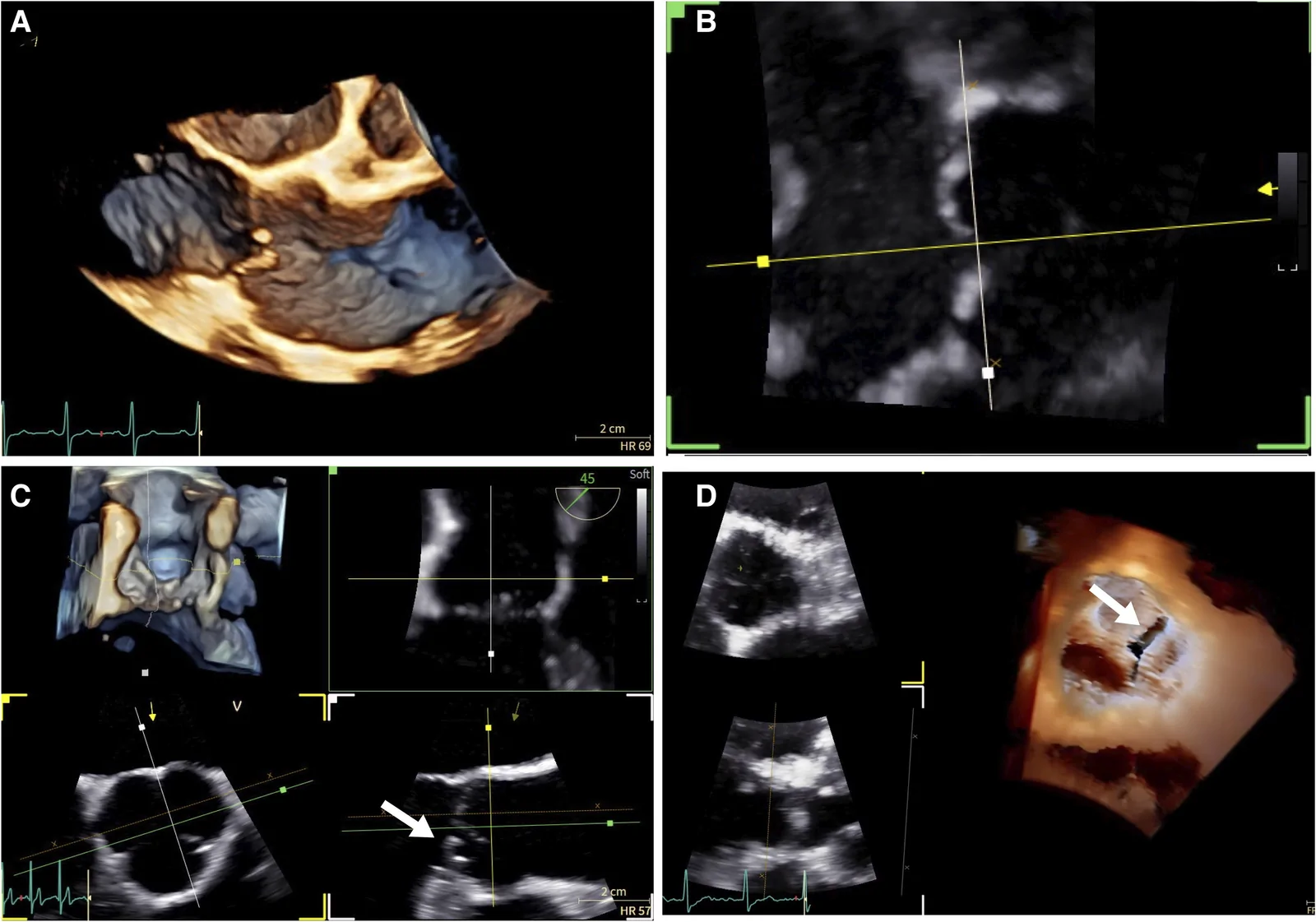
Mechanisms of aortic regurgitation (AR) according to El Khoury’s classification. Panel *A*: 3D ‘long-axis’ view of a patient with type I AR with dilatation of the VBR, aortic root, sinotubular junction, and tubular ascending aortic aneurysm. Panel *B*: Multiplanar reconstruction (MPR) of the same patients showing reduced coaptation height and symmetrical reduction in effective height. Panel *C*: MPR of a patient with complete prolapse of the right cusp (white arrow) in a tricuspid valve. Note that the effective height is < 0 mm. Panel *D*: 3D ‘en face’ view with transillumination modality obtained during a transthoracic exam. The light in aorta highlights the retraction of the left cusp (white arrow).

In Type I AR according to El Khoury’s classification, disproportionate dilatation of the STJ and TAA (Type 1a), of the SoV and STJ (Type 1b), or of the VBR (Type 1c) relative to the cusp size, causes central AR (*Figure 4A*). STJ dilatation plays a central role in Type 1a and 1b: stretching of this region and the adjacent commissures is responsible for cusp tethering.^13^ In terms of heights, an increase in the STJ/VBR diameter ratio above 1.2 (Type 1a and 1b) causes a reduction in cH, while VBR dilatation—isolated (Type 1c) or combined with aortic dilatation—causes a reduction in cH and a symmetrical reduction in eH (*Figure 4B*).^23,24^ Progressive dilatation of the STJ and/or TAA may lead to an increase in free margin length and gH.^22^ This is a compensatory mechanism aiming to keep the coaptation surface as good as possible, but ultimately fails when AR ensues. Cusp perforation is also part of this group (Type 1d).

In Type II AR, prolapse is defined as a reduction of the eH of one cusp compared with the other two. Reduced eH may result in incomplete (marginal) prolapse if eH is <9 mm, but the cusp remains above the VBR plane, or complete prolapse if eH is < 0 mm (below the plane of the VBR) (*Figure 4C*).^25^ The most common cause of prolapse is elongation of the cusp-free margin, although it may also result from ruptured fenestration, commissural disruption, or surgical distortion.^26^

In type III AR, retraction is defined as the evidence of gH ≤16 mm in TAV and gH ≤18 mm in the non-fused cusp in BAV.^20^ Organic retraction is due to thickening, calcification, fibrosis, destructive endocarditis, or rheumatic diseases (*Figure 4D*).

These mechanisms may coexist, making anatomical and functional assessment more complex. Therefore, the use of 3D TOE with MPR is pivotal for an accurate diagnosis.

### A 3D step-by-step and repair-oriented approach to aortic regurgitation

**Figure 5:**
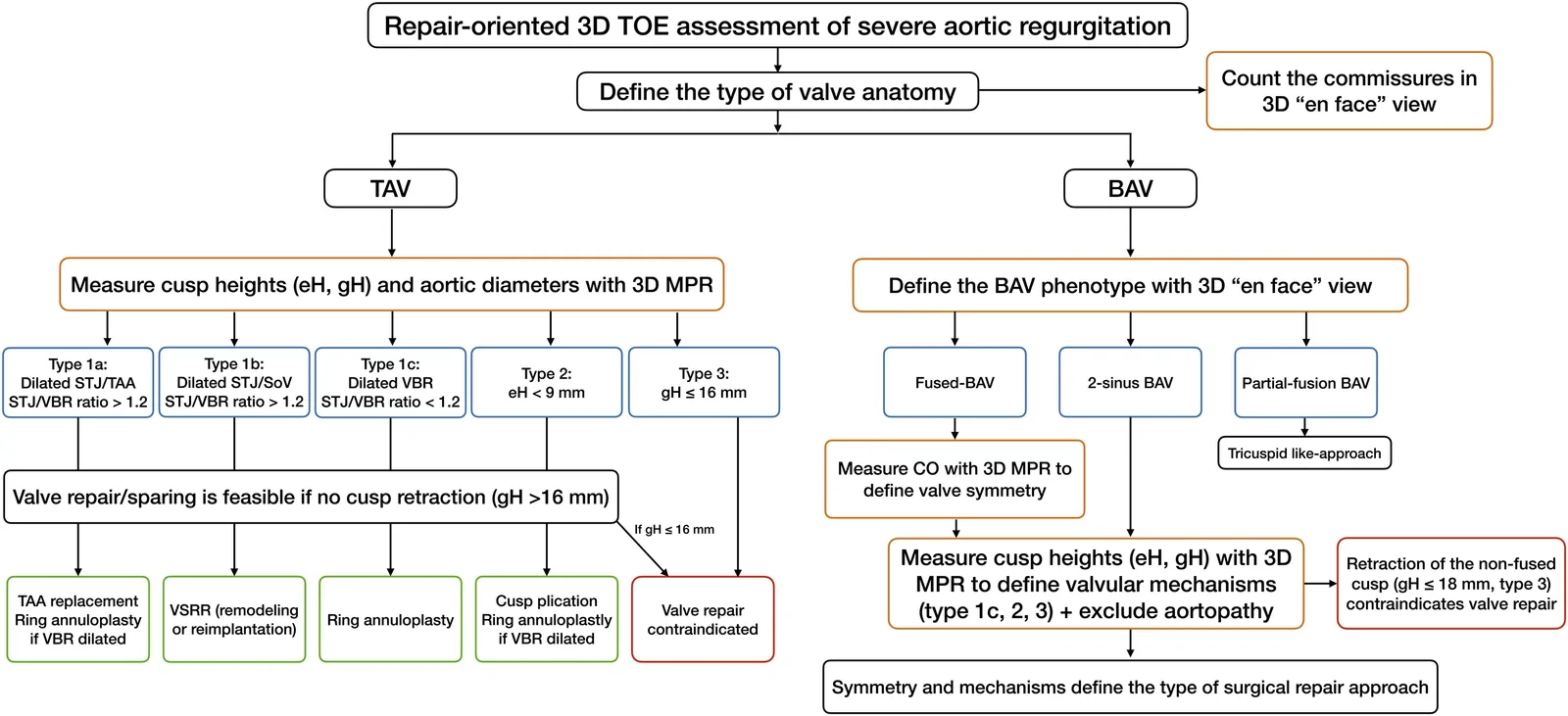
Proposed flowchart for repair-oriented 3D transoesophageal echocardiographic (TOE) assessment of severe aortic regurgitation.

When approaching a significant AR, the imaging cardiologist’s perspective should be focused on the possibility of repairing or sparing the valve (*Figure 5*). The first aspect to define is the type of valve anatomy. The 3D TOE en face view represents the ideal approach for this purpose, as it accurately highlights the commissures.

In TAV, the second step is the identification of the AR mechanism. To achieve this, 3D TOE with MPR is pivotal to measure cusp heights and aortic diameters. In Type 1a and 1b AR, exclusion of cusp intrinsic pathology (fibrosis, calcification, perforations) and retraction (defined as gH of ≤16 of at least one cusp),^2,20^ may allow AR to be treated without replacing the AV. Thus, the ascending aorta replacement is the gold standard treatment for Type 1a. An additive external ring sub-valvular annuloplasty is considered in experienced centres when the VBR is dilated to avoid the risk of AR recurrence.^26^ In Type 1b AR, VSRR techniques—either Yacoub (remodelling) or David (reimplantation)—should be proposed. VSRR may induce cusp prolapse; therefore, the intraoperative 3D MPR assessment should focus on eH measurement to determine whether a central plication technique should be added to reduce the free margin length of the prolapsing cusp.^27,28^ Type 1c AR is rarely isolated, being often combined with cusp prolapse. The dimensions of the VBR (diameters, circumference, and area) are appropriately assessed using 3D MPR during either a TTE or, preferably, a TOE exam. A surgical annuloplasty represents the treatment of choice for VBR dilatation (VBR ≥25 mm). Several annuloplasty techniques have been proposed and are currently adopted according to individual centre experience: external ring annuloplasty (combining two rings placed at the VBR and STJ levels to restore an appropriate STJ/VBR ratio),^29^ suture annuloplasty,^30^ internal ring annuloplasty,^31^ or combination of external and internal annuloplasty at the VBR and STJ levels.^32^ Annuloplasty is expected to increase the cH, without significantly modifying the eH.^26^

Type II AR is the most frequent intrinsic AR mechanism in TAV, excluding aortic aneurysms. In a large retrospective registry, prolapse accounted for 45.9% of the aetiologies in patients with TAV and significant AR undergoing surgery; the right cusp was the most frequently involved cusp (83.1%), followed by the non-coronary cusp (10.3%).^2^ 3D TOE with MPR assessment should include measurement of eH in all cusps to identify a prolapse (complete or incomplete), as well as gH to exclude retractions (gH ≤16 mm).^2,20^ The presence of cusp retraction generally precludes durable valve repair due to the high risk of suboptimal long-term results.^2,33^ To complete the pre-operative evaluation, VBR, coronary height, and aortic diameters should be measured using 3D TOE with MPR. Cusp fenestrations are congenital gaps in the peri-commissural zone, beneath the free margin. Most of them do not cause insufficiency or result only in mild AR, visible as a small jet near the commissures on the 3D colour en face view. However, they can be involved in the prolapse mechanism when a rupture of the free margin occurs at the level of the fenestration.^26^ 3D TOE long-axis view can show the ruptured fenestration. In cases of fragile, multiple and/or ruptured fenestration, AV repair is complex and may require a pericardial patch.

As stated before, Type III AR represents a contraindication to valve repair for the risk of limited durability.^2,33^

**Figure 6:**
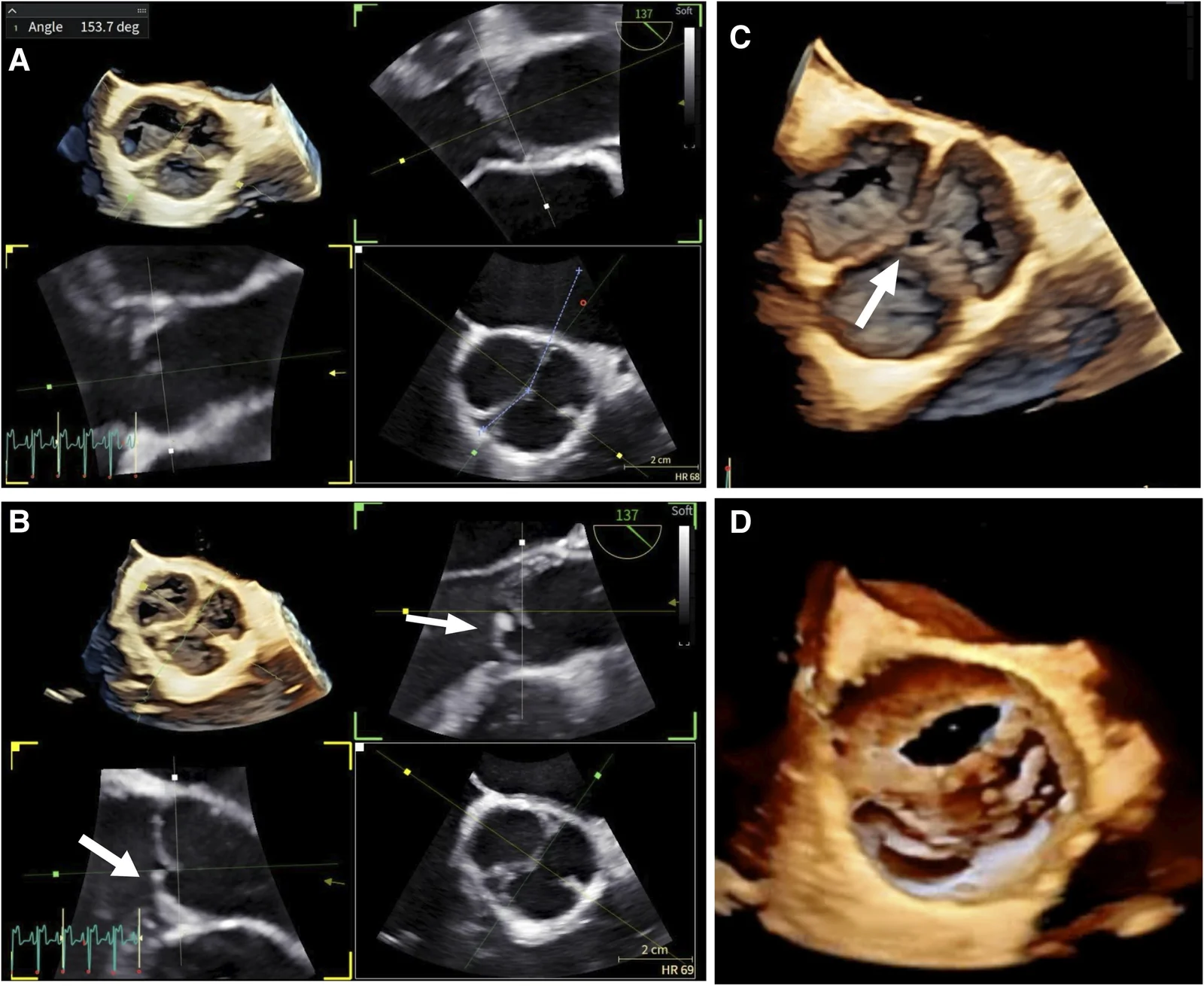
3D assessment of a bicuspid aortic valve. Panel A: Measurement of the commissural orientation (CO) through 3D multi-planar reconstruction (MPR) in diastole. This valve is asymmetrical (CO = 153.7°). Panel B: 3D MPR used to assess cusp heights along the entire coaptation line between the two cusps. This valve shows prolapse of the fused cusp (white arrow). Panel C: 3D ‘en face’ view showing retraction of the cusp free edge at the raphe level. Panel D: 3D ‘en face’ view with transillumination modality of a unicuspid aortic valve with a unique postero-lateral commissure (valve oriented as in the mid-oesophageal short-axis view).

In BAV, the first step is to define the valve phenotype. An international nomenclature has been introduced to provide a standardized and repair-oriented classification based on imaging findings.^34^ The 3D TOE en face view offers the best perspective for classifying a BAV according to this nomenclature: fused-BAV, 2-sinus BAV, or partial-fusion BAV (forme fruste).^34^ In fused-BAV type, the most frequent phenotype (90–95% of the cases), three aortic sinuses and two cusps (usually different in size) are present; a raphe is common but may not be visible. The 3D en face view is particularly useful in young patients, in whom the raphe may be thin and only mildly fibrotic, making it difficult to be detected with 2D imaging. In 2-sinus BAV (5–7%), the 3D en face view shows two aortic sinuses and two cusps (similar in size) without a raphe. The partial-fusion form (prevalence unknown) shows three aortic sinuses with a mini-raphe between two cusps. A critical aspect for AV repair, closely linked to this classification, is valve symmetry in the fused-BAV phenotype. Symmetry is defined by commissural orientation (CO), the angle between the two commissures, measurable through 3D TOE with MPR in diastole (*Figure 6A*). Accordingly, fused-BAV can be classified as symmetrical (CO: 180°–160°), asymmetrical (CO: 160°–140°), or very asymmetrical (CO: 140°–120°).^8^ Valve symmetry influences the repair technique: central plication in symmetric valves, raphe direct closure in asymmetric valves (to increase the CO towards 180°), and tricuspid-like repair for very asymmetric valves.^8^ Importantly, asymmetrical valve configuration negatively affects freedom from reoperation after AV repair.^35^

The second step in BAV assessment is identifying the mechanism of AR. The most common mechanism in BAV is the prolapse of the fused cusp, or of one of the cusps in the 2-sinus phenotype (*Figure 6B*). Another specific BAV mechanism in asymmetrical valves is the retraction of the free edge at the raphe level (*Figure 6C*).^34^ 3D TOE with MPR is used to assess cusp heights along the entire coaptation line between the two cusps to accurately define the mechanism. The retraction of the non-fused cusp, defined as gH ≤18 mm, represents a contraindication to AV repair (‘surgical cut-off’)^20,25^ 3D VBR measurement is mandatory because BAV usually has a dilated annulus. Therefore, the previously described surgical annuloplasty techniques are commonly applied during a BAV repair.^29–32^ Additional relevant measurements obtained by 3D TOE with MPR include coronary ostial heights and aortic diameters.

Unicuspid AVs (*Figure 6D*) often combine stenosis and regurgitation, therefore, 3D AV area planimetry should be performed before considering valve repair. Valve calcification, which usually involves the region corresponding to the right cusp, should also be carefully excluded.^36^

### 3D assessment of aortic stenosis and for the planning of aortic valve replacement

**Figure 7:**
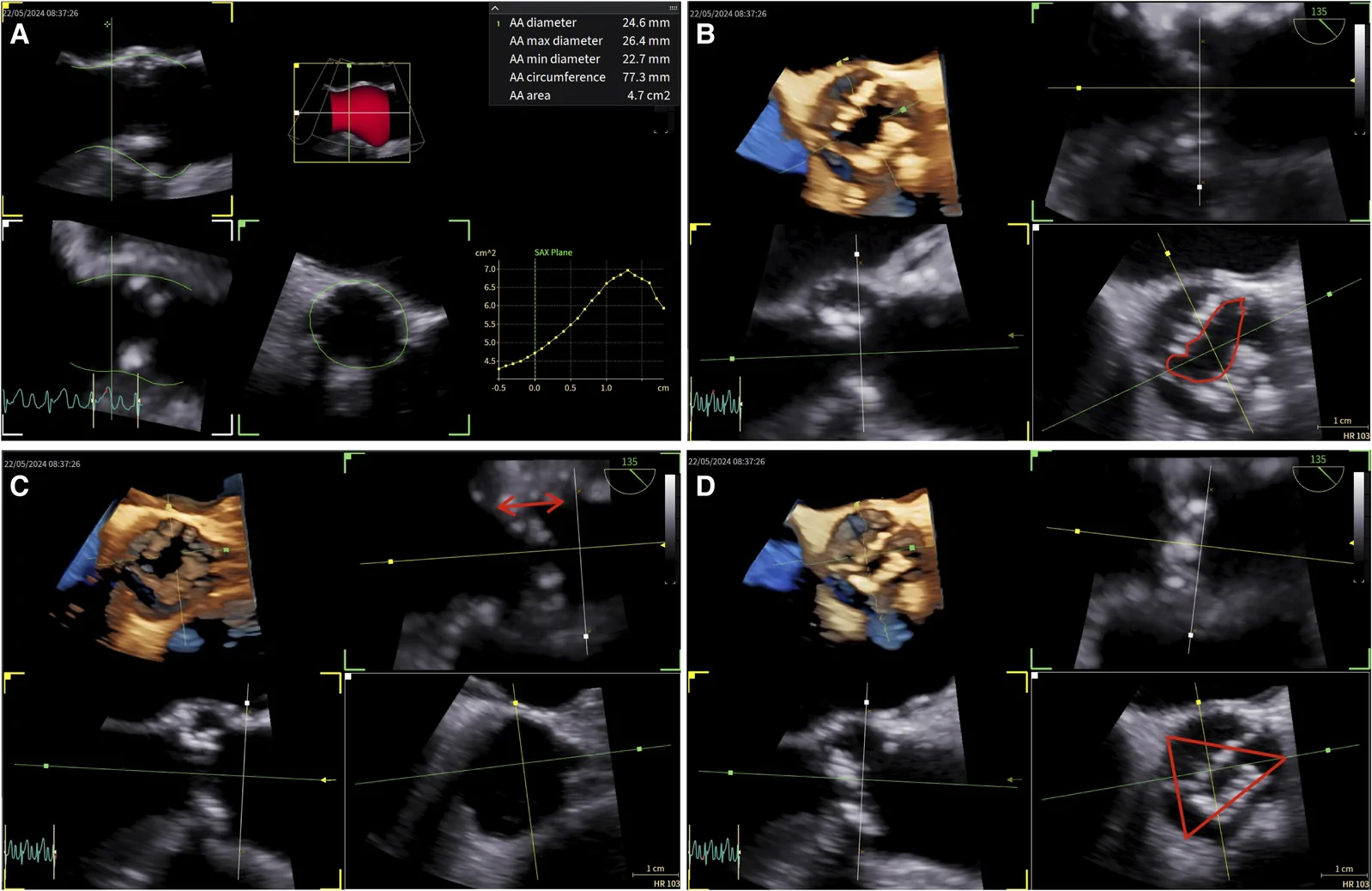
3D assessment of aortic stenosis and for the planning of aortic valve replacement. Panel *A*: Semi-automated assessment of left ventricular outflow tract (LVOT) area using 3D TOE. Panel *B*: Aortic valve area (AVA) by planimetry obtained by 3D TOE with multiplanar reconstruction (MPR). Panel *C*: assessment of left coronary artery ostial height using 3D MPR. Panel *D*: measurement of the sinuses of Valsalva (sinus-to-sinus diameters) using 3D MPR.

In the assessment of a patient with aortic stenosis (AS), TTE may reveal complex or borderline scenarios: the aortic valve area (AVA), calculated using the continuity equation, may be at the threshold of severity; apparently severe low-gradient AS may be discordant with the degree of systolic valve opening; stroke volume estimation using Doppler method may be unreliable in cases of sub-optimal imaging or flow acceleration in the LVOT or may differ significantly from volumetric measurements. 3D TOE may help clarify these situations. Manual and semi-automated assessment of LVOT area using 3D TOE has been demonstrated to reduce the proportion of patients in a low-flow state, thereby reducing the proportion of patients with low-gradient AS and pathological AVA^37^ (*Figure 7A*). In the same study, LVOT area measured by 3D TOE was slightly smaller than that measured with contrast-enhanced CT, thus generating a difference in AVA estimation.^37^ Furthermore, 3D TOE with MPR allows direct measurement of the 3D AVA by planimetry (*Figure 7B*). The AVA planimetry is larger than AVA derived from the continuity equation, as the latter reflects the effective flow area distal to the geometric orifice.

3D TOE can be particularly useful in the planning of aortic valve replacement, both transcatheter and surgical. In the context of TAVI planning, 3D TOE may serve as an alternative when contrast-enhanced CT is contraindicated or unfeasible, such as in urgent procedures or in patients with contrast allergy, irregular rhythm, or significant chronic kidney disease. Manual, semi-automated, or fully automated methods enable measurement of the VBR (diameters, perimeter, and area) to decide the appropriate prosthesis type and size. Compared with contrast-enhanced CT, manual measurements showed larger underestimation of the VBR dimensions (10–20%) than semi-automated or automated approaches (1–3.5%).^38,39^ All three methods showed only a moderate agreement in prosthesis sizing compared with CT.^39^ 3D TOE can also complement peri-procedural TAVI risk stratification through measurement of coronary heights and aortic root dimensions (*Figure 7C*). In particular, the dimensions of the SoV are crucial for assessing the risk of coronary obstructions (*Figure 7D*).

For planning a surgical AV replacement, 3D assessment of the VBR can help prevent patient-prosthesis mismatch.^40^ When a small annulus relative to body surface area is identified, annular enlargement procedures may be proposed.^9^ However, in all these applications, 3D TOE has the limitation of potentially sub-optimal imaging in heavily calcified AVs due to blooming artefacts.

### Limitations

Despite its growing clinical role, 3DE remains dependent on image quality and operator expertise. To obtain a high-resolution 3D image during a TOE exam, 2D imaging quality and technical parameters (depth, sector width) should be optimized to achieve a volume rate of at least 10–15 volumes per second in single-beat modality. Whenever it is not feasible, multi-beat modality can significantly enhance volume rates, providing that the patient has a stable heart rhythm and is able to hold the breath. Despite correct 3D dataset acquisition, artefacts may still limit the possibility of reconstructing the AV in 3D.^41^ Dropouts are common artefacts on the AV and appear as a lack of valvular tissue. They are frequently evident in diastole because the ultrasound beam direction is parallel to the body of the AV cusp. Inexperienced operators may confound dropouts with valvular perforations. Acoustic shadowing artefacts, generated by highly reflective structures, may limit visualization in heavily calcified valves or prostheses. This frequently limits the possibility of precisely measuring AVA by planimetry with MPR in severe AS. All these described technical limitations and drawbacks of 3D imaging are more evident in the TTE setting.^42^ Dropout artefacts still strongly limit AV visualization in 3D TTE. Furthermore, the measurement of the geometric parameters for a valve repair assessment (eH, gH, cH) is not reliable in TTE. Regarding operator expertise, imaging acquisition, post-processing, and MPR require specific training. Finally, although automated software solutions have improved reproducibility, standardization across vendors and validation against surgical measurements remain areas for further investigation.

## Conclusions

3DE, especially in the TOE setting, has become an essential imaging modality by providing comprehensive anatomical and pathophysiological information on the AV and aortic root complex, thereby facilitating optimal treatment selection. In particular, the feasibility of valve repair or valve-sparing surgery should be systematically assessed when evaluating patients with AR. Moreover, 3D TOE provides additional insights in the assessment of AS, plays a pivotal role in the planning of surgical valve replacement procedures, and serves as an alternative imaging method for planning transcatheter procedures when contrast-enhanced CT is contraindicated or unfeasible.

## Data Availability

No new data were generated or analysed in support of this research.

## Lead Author Biography

Prof. Patrizio Lancellotti, MD, PhD, is Head of the Cardiology Department at CHU Sart Tilman, University of Liège, Belgium. He is an internationally recognized expert in cardiovascular imaging, valvular heart disease, and structural heart interventions. He is Past-President of the European Association of Cardiovascular Imaging, Past-President of the Belgian Society of Cardiology, and Past-Chair of the ESC Council on Valvular Heart Disease. He serves on several editorial boards and has authored more than 960 scientific publications and several textbooks.
